# Accumulation Kinetics of Three Scirpentriol-Based Toxins in Oats Inoculated *in Vitro* with Isolates of *Fusarium sporotrichioides* and *Fusarium poae*

**DOI:** 10.3390/toxins3050442

**Published:** 2011-05-09

**Authors:** Margit Schollenberger, Hans-Martin Müller, Melanie Liebscher, Claudia Schlecker, Melanie Berger, Wilfried Hermann

**Affiliations:** 1 Institute of Animal Nutrition, Hohenheim University, Emil-Wolff-Str. 10, 70599 Stuttgart, Germany; Email: hans-martin.mueller@t-online.de (H.-M.M.); m.liebscher@uni-hohenheim.de (M.L.); ins450@uni-hohenheim.de (C.S.); m.berger@uni-hohenheim.de (M.B.); 2 Experimental Station Ihinger Hof, Hohenheim University, 71272 Renningen, Germany; Email: wilfried.hermann@uni-hohenheim.de

**Keywords:** * Fusarium poae*, *Fusarium sporotrichioides*, oats, scirpentriol toxins, sequential accumulation, toxigenicity, trichothecenes

## Abstract

Autoclaved oats were inoculated with a strain of *Fusarium sporotrichioides* or* Fusarium poae. *Moisture content of oats after inoculation was at 38%, incubation took place in standing culture at 28 °C. The A-type trichothecenes, 4,15-diacetoxyscirpenol (4,15-DAS), 15-monoacetoxyscirpenol (15-MAS), and scirpentriol (SCIRP) were analyzed by GC/MS. For each strain, three culture flasks were harvested at 2–3 day intervals starting immediately after inoculation. Total incubation time was 42 days (*F. poae*) and 56 days (*F. sporotrichioides*). Following peak accumulation, 4,15-DAS decreased below the detection limit for both strains, 15-MAS decreased below this limit for the isolate of *F. sporotrichioides*, for the isolate of *F. poae *it decreased to a level markedly below the peak value. SCIRP, after having peaked, decreased to some extent for the strain *F. sporotrichioides*, with a significant (*P* = 0.0029) negative linear regression of toxin content against culture age during this period*. *The content of 15-MAS, and in part also of 4,15-DAS, decreased along with an increase of SCIRP. This sequential accumulation pattern suggests the successive induction of esterases deacetylating 4,15-DAS and 15-MAS, as well as of enzymes involved in the metabolization of the parent alcohol, SCIRP. The results may explain, at least in part, the somewhat higher incidence in naturally contaminated compounds reported in the literature for SCIRP compared to 4,15-DAS and 15-MAS.

## 1. Introduction

*Fusarium* species play an important role as plant pathogens, which cause a wide range of diseases in a diversity of host plants with substantial economic losses on a worldwide basis. Many species are able to produce toxic secondary metabolites [1,2]. The present paper describes the increase and decrease of the toxins, 4,15-diacetoxyscirpenol (4,15-DAS), 15-monoacetoxyscirpenol (15-MAS) and scirpentriol (SCIRP) in oats inoculated with a strain of *Fusarium sporotrichioides* or *F. poae*. These toxins are members of the scirpentriol subgroup of A-type trichothecenes, with two deacetylation steps leading from 4,15-DAS via 15-MAS to the parent alcohol, SCIRP. 

The aim of the present investigation was to further the techniques of toxigenicity studies. So far, the ability of a *Fusarium* strain to produce toxins has been studied mostly with batch cultures harvested only once for analysis. This has recently been reviewed for toxins of the scirpentriol subgroup of A-type trichothecenes and a variety of *Fusarium* strains [3]. The toxin spectrum may change along with culture age [4,5,6,7,8,9,10,11], including the complete disappearance of a toxin after some time. This has been reported for the B-type trichothecenes nivalenol and deoxynivalenol and their acetylderivatives [4,5,6,7,8], as well as for the A-type trichothecenes, DAS, HT-2 toxin, T-2 toxin, and neosolaniol [10,11]. Therefore, if a toxin is not detected using the one-harvest technique this may be the result of too early or too late harvest and not a genetically determined inability of the strain investigated or environmental conditions. Likewise, the relation of toxin contents produced simultaneously in a *Fusarium* culture [12] may depend on the length of incubation. In the present study, aimed at the ability of two *Fusarium* species to produce three scirpenol toxins, a multiple-harvest technique was used which started immediately after inoculation and included short time intervals. To our knowledge, kinetic analysis determined in this way has not yet been described for any toxins of this subgroup.

Environmental conditions influencing toxin production may imply the type of medium on which the culture is grown. So far, research dealing with the *in vitro* formation of scirpentriol toxins has been carried out using synthetic or semisynthetic media, among cereals, barley, rice, maize, wheat, corn [3] or a mixture of wheat, oats and barley [12]. For the present study, oats have been chosen as substrate because their infestation with a variety of *Fusarium* species as well as contamination with trichothecenes under natural conditions has been widely described [12,13,14,15,16]. Compared to wheat and barley, oats contained a higher incidence of type-A trichothecenes in Norwegian cereals [16]. Recently, 13 out of a total of 16 *Fusarium* toxins examined have been found in naturally infected oat samples in Germany [17]. Up to 10 toxins co-occurred in the same sample including six A-type and three type-B trichothecenes as well as ZEA [17].

*F. sporotrichioides* has a wide distribution in soils and plants, particularly in cereal grasses in cooler climates, and has been associated with maize ear rot and head blight of small grain cereals in Canada, the United States and Europe [18]. *F. poae* is a fungus of increasingly recognized importance, with DAS, MAS and SCIRP produced as main trichothecenes [19]. It has been isolated from a wide variety of hosts, particularly cereals and grasses, in temperate and cold areas worldwide [20]. *F. poae *has been associated with head blight of small grain cereals in North America and Japan, and is a major component of the head blight complex of barley, oats, and wheat in Europe. Strains of both species have been isolated repeatedly from oats [1,21,22] whereas a production of trichothecenes on oats *in vitro*, to our knowledge, has not yet been described.

## 2. Material and Methods

### 2.1. Substrate, Inoculation, Incubation, Sampling Device

Oat kernels were purchased on the retail market and ground to a particle size of about 2 mm. This batch has been previously analyzed according to Schollenberger *et al.* [23,24] to assess for the absence of the toxins under investigation. The ground oat material was moistened to a water content of 33 %, and portions of 65 g together with 50 g of boiling stones (diameter 6 mm, VWR, Darmstadt, Germany), used to avoid agglomeration, were placed in 500 mL Erlenmeyer flasks. These were plugged with cotton, covered with aluminium foil and autoclaved for 20 min at 120 °C. After cooling down, the vessels were shaken to loosen the content and inoculated with 5 ml of mycelium suspension per flask, thus achieving a moisture content of 38%. After inoculation, vessels were plugged with cotton, and parafilm was used to fix the plug on the flask. For toxigenicity studies of *Fusarium* species on cereals, water addition up to 33–38% has been reported [25,26].

A strain of *F. sporotrichioides Sherbakoff var minus Wollenweber *(DSMZ 62425) and of *F. poae Wollenweber* (DSMZ 62376), both originally isolated from oats and grown on potato-dextrose-agar (PDA) slants, were purchased from the German Collection of Microorganisms and Cell Cultures (Braunschweig, Germany). PDA was made from 1000 mL of boiled extract of 200 g potato tubers, 20 g dextrose and 15 g agar. The content of a stock culture was suspended in 25 mL of autoclaved water using a high speed blender. Four 500 mL Erlenmeyer flasks containing 50 mL SNY medium were inoculated with 2.5 mL of this stock suspension and shaken on a rotary shaker at room temperature for 48 h in the dark. SNY- medium contained (per liter): 1 g of KH_2_PO_4_, 1 g of KNO_3_, 0.5 g MgSO_4_ × 7 H_2_O, 0.5 g KCl, 0.2 g glucose, 0.2 g saccharose, 10 g yeast extract, brought to pH 5.5 to 5.8 with KOH. The resulting mycelia were macerated, the contents of 4 flasks combined and mixed with 300 mL of autoclaved water. 5 mL of this mycelium suspension per vessel were used for the inoculation of oats. 

Inoculated flasks were maintained in the dark in an incubator at 28 °C. This temperature has been described to be within the optimal range of *F. sporotrichioides* and *F. poae *[2]. For each strain, three culture bottles were harvested at 2–3 day intervals starting immediately after inoculation. Total incubation was for 42 days (*F. poae*) and 56 days (*F. sporotrichioides*). The trial using an isolate of *F. poae* was done first, for the following experiment using an isolate of *F. sporotrichioides.* The total incubation time was prolonged to allow for a better observation of SCIRP kinetics.

### 2.2. Analysis of Mycotoxins

Standard substances were purchased from Sigma (Deisenhofen, Germany). The content of each flask was pulverized and transferred with 435 mL of a mixture of acetonitrile/water (75/25, v/v) (10 g of oat dry matter/100 mL solvent) into a 1 L screw-capped bottle. The content was stirred vigorously using a magnetic stirrer at high level for 1 h and filtered through a folded filter. 63 mL of the filtrate were extracted with hexane as described elsewhere [23]. This was followed by clean-up using a florisil and a cation exchange cartridge (both from Varian, Darmstadt, Germany). Elution from the cation exchange cartridge was done using a mixture of acetic acid/methanol (60/40, v/v). Derivatization was performed with trifluoraceticanhydride and verrucarol was used to control derivatization efficiency [24]. Separation and quantitation was by GC/MS using a Magnum Ion Trap system (Finnigan, Bremen, Germany) consisting of a Finnigan MAT ion-trap detector ITD 800 interfaced to a Varian Star 3400 CX gas chromatograph. A DB-5 MS phase (30m × 0.25 mm, film thickness 0.25 µm) (J&W Scientific, Folsom, CA, USA) was used as a capillary column. The carrier gas was helium 5.0. The temperature of the injection port was 260 °C and injection volume was 1 µL. The temperature program of the gas chromatograph was at: 140 °C (2 min)–7 °C/min–275 °C (2 min)–30 °C/min–290 °C (5 min). The temperature of the transfer line was adjusted to 270 °C. The mass spectrometer was operated in the chemical ionisation mode using isobutane (3.5) as reagent gas. The temperature of the ion trap was 190 °C. The maximum ionisation time was 1500 µs, the maximum reaction time 80 ms, ionisation level 25 u, the reaction level 40 u, reagent ion eject level 250 u, the reagent ion eject adjust 100 %, and the reagent reaction time 9000 µs. The scan range selected for full-scan data was 280–650 u [23]. 

Toxins were identified by comparing spectra with spectra obtained from the commercial standards. Detection limits were assessed at a signal to noise ratio of 3:1 and were at 10, 5, and 20 µg/kg for 4,15-DAS, 15-MAS and SCIRP, respectively. 

### 2.3. Curve Fitting and Statistics

**Figure 1:**
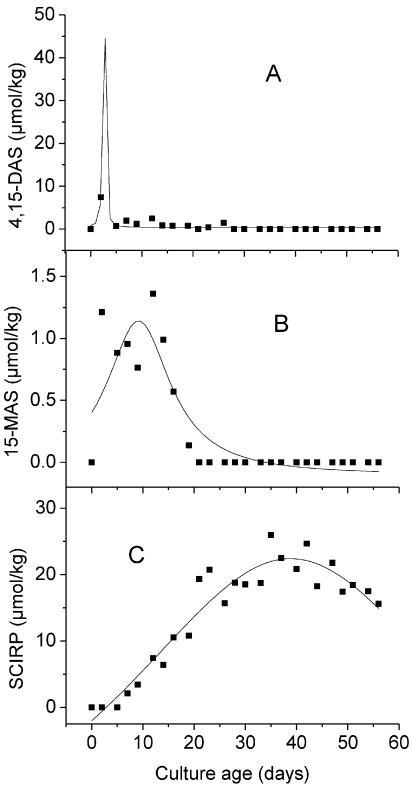
Time course of 4,15-diacetoxyscirpenol (**A**), 15-monoacetoxyscirpenol (**B**) and scirpentriol (**C**) in batch cultures of *F. sporotrichioides *in oats (initial moisture content 38%, incubation at 28 °C). Data points represent the average of three culture flasks. The Lorentz model was used for non-linear curve fitting (see Material and Methods).

**Figure 2:**
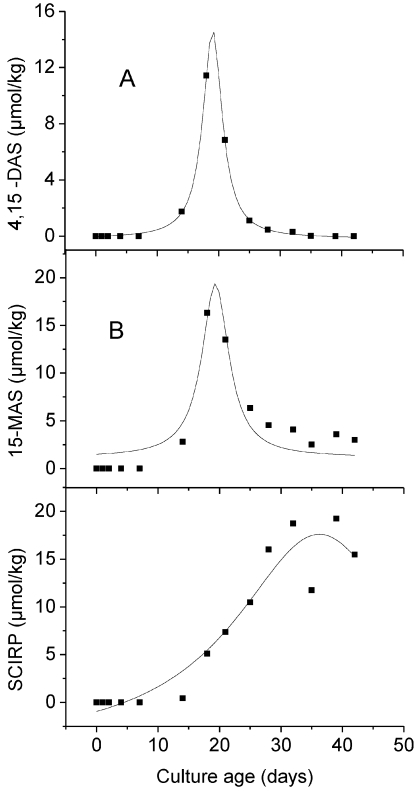
Time course of 4,15-diacetoxyscirpenol (**A**), 15-monoacetoxyscirpenol (**B**) and scirpentriol (**C**) in batch cultures of *F. poae *in oats (initial moisture content 38%, incubation at 28 °C). Data points represent the average of three culture flasks. The Lorentz model was used for non-linear curve fitting (see Material and Methods).

Curve fitting was carried out using the program Origin 7 G SR 1 (OriginLab, Northampton, USA). Generally, a fitting procedure seeks to find those parameter values that minimize the deviations between the observed and the expected y values, based on a certain model for the definition of the latter. For the present study, we used the nonlinear Lorentz model, which yielded a good agreement between the data and the model curves (Figure 1, Figure 2). This was documented amongst others by high correlation coefficients. In nonlinear models, one cannot usually solve the equation for the parameters, and various iterative procedures are used instead. Origin uses the Levenberg-Marquardt algorithm to iteratively adjust the parameters to get the minimum of chi-square value. For a description of this method see Press *et al. *[27]. The significance of the decrease of SCIRP following peak accumulation in cultures of *F. sporotrichioides* (Figure 1) was also tested using the linear regression model provided by Origin.

## 3. Results

Mycelial growth had started visibly in all flasks within the first days following inoculation in the oat medium. With the isolate of *F. sporotrichioides*, an exothermic reaction was observed for the first week. During growth of both strains, water accumulated on the surface of the mycelium, beginning at day 6 after inoculation and reaching a maximum at about day 20. After about day 28, a dehydration could be assumed as drops of water disappeared from the surface. 

The time course of the identification of the three scirpentriol mycotoxins in batch cultures of *F. sporotrichioides* and *F. poae* using oats as substrate is depicted in Figure 1 and Figure 2, with the non-linear curve fitting based on mean toxin contents. For both strains, the content of all three mycotoxins increased to a maximum value. Following peak accumulation 4,15-DAS decreased below the detection limit for both strains; the same was true for 15-MAS with the isolate of *F. sporotrichioides*, while this toxin decreased to about 16 % of the maximal value for the strain of *F. poae*. After accumulation, SCIRP decreased to about 60 % of the maximal value for the isolate of *F. sporotrichioides*.

The high quality of non-linear fitting based on the Lorentz model is indicated by high correlation coefficients. Thus, for the isolate of *F. sporotrichioides*, the R-square value was at 0.8078 for 4,15-DAS, 0.7770 for 15-MAS, and at 0.9212 for SCIRP; for the strain of *F. poae* it was at 0.9995 for 4,15-DAS, 0.8858 for 15-MAS, and at 0.9186 for SCIRP. This indicates a decrease of toxin content following peak accumulation for both *Fusarium* strains, not only for 4,15-DAS and 15-MAS but also for SCIRP. The decrease of SCIRP following peak accumulation in cultures of the isolate of *F. sporotrichioide*s was also demonstrated by a highly significant (*P* = 0.0022) negative linear regression of SCIRP contents against culture age during the period of 35–56 days after inoculation.

The iterative non-linear curve fitting yielded nearly the same incubation time up to peak accumulation, independent on whether the mean or single toxin contents were introduced into the model. The R square value of the fitting based on single toxin contents was somewhat lower compared to the fitting based on mean values. For the strain of *F. sporotrichioides* it was at 0.7383, 0.6974 and 0.7203 for 4,15-DAS, 15-MAS and SCIRP respectively, while corresponding values for the isolate of *F. poae* were at 0.5304, 0.4706 and 0.4798. 

The non-linear curve fitting revealed that the kinetics of the three scirpentriol toxins followed a sequential pattern for both strains. It was characterized in particular by successive peaking in the order 4,15-DAS, 15-MAS and SCIRP. Thus, for the strain of *F. sporotrichioides*, the non-linear fitting model yielded the peaking of 4,15-DAS, 15-MAS and SCIRP at 2.6 ± 1.4, 9.2 ± 0.9, and 38.8 ± 1.1 days after inoculation respectively. For the isolate of *F. poae*, the corresponding incubation times were 19.0 ± 0.02, 19.3 ± 0.3, and 36.2 ± 1.9 days. For both strains 4,15-DAS and 15-MAS decreased along with the increase of SCIRP (Figure 1, Figure 2). 

## 4. Discussion

The present study describes in detail, for the first time, the increase and decrease of selected scirpentriol toxins in *Fusarium* batch cultures. For both strains tested, concentrations of the acetylesters 4,15-DAS and 15-MAS declined markedly with a complete disappearance of 4,15-DAS in cultures of *F. sporotrichioides* and *F. poae* and 15-MAS in the isolate of *F. sporotrichiodes* after some time (Figure 1, Figure 2). As mentioned above, a complete *in vitro* disappearance of toxins has been observed under certain culture conditions for some type B- and type-A trichothecenes [4,5,6,7,8,10,11]. Thus, for toxigenicity studies, it should be considered that the toxin spectrum may vary with the age of the *Fusarium* cultures and may include the complete disappearance of a toxin. Therefore, the use of several sampling dates instead of a single harvest time should be taken into consideration for those studies [3]. The relationship of toxins produced simultaneously in a *Fusarium* culture also varied with water activity/temperature combinations after a certain incubation period [12].

The kinetics of the three scirpentriol toxins as described in the present study may be the result of numerous factors. Environmental effects, including substrate, temperature, and water, influence the amount as well as the type of toxins produced [5,8,12]. The substrate moisture content in this study was not kept constant. Water appeared visibly during the first period of incubation, probably resulting from respiration. During this phase a water activity of 1.0 can be assumed, afterwards visible water disappeared. It cannot be excluded that the accumulation kinetics of the three assayed scirpentriol toxins (Figure 1, Figure 2) were impacted by changes in the water activity of the substrate. To our knowledge, studies on the effect of this factor on the formation and disappearance of the threescirpentriol toxins are not available. Conditions encountered in the field are probably approximated by a variable better than a constant water activity.

A second viewpoint refers to the activity of enzymes involved immediately in the formation and metabolization of the three scirpentriol toxins. Generally, it is assumed (see amongst others Desjardins, [28]) that trichothecenes, in common with many other fungal natural products, are not required for normal growth. Genes of trichothecene biosynthetic pathway in *Fusarium* species therefore are assumed to be expressed not constitutively but rather in response to developmental and environmental signals. Trichothecene biosynthetic genes encode enzymes that catalyze acetylation and deacetylation of trichothecenes, as well as other steps [18]. Thus, the sequential accumulation of 4,15-DAS, 15-MAS and SCIRP may reflect the inductive, and therefore successive, formation of esterases catalyzing the two steps from the esters to the parent alcohol SCIRP, with the appearance of substrate as the inducing event. This hypothesis is consistent with the observation of Park and Chu [29] that the production of deacetylated trichothecenes in *F. sporotrichioides* cultures on a liquid medium was associated with an increased total activity of fungal esterases. The successive formation of esterases apparently did not exclude the simultaneous presence of precursor and product during certain phases of fungal development (Figure 1, Figure 2). An inductive and hence successive formation of esterases may explain also the sequential accumulation of *Fusarium* toxins described in the literature, e.g., the accumulation of NIV following that of FUS-X by *Gibberella zeae* [7], and the accumulation of DON succeeding that of ADON by *F*. *graminearum *[4,5,8,30]. An induction may have occurred also of enzymes involved in the formation of 4,15-DAS as well as in the decrease of SCIRP.

For the strain of *F. sporotrichioides*, a decrease of the parent alcohol SCIRP following peak accumulation has been indicated both by the high R square value of non-linear fitting, as well as by a highly significant negative linear regression between toxin content and culture age during the period of decrease. To our knowledge, this is the first proof of a decrease of SCIRP in *Fusarium* cultures. For inoculated wheat plots, a decline in the field is also described for the parent alcohol DON [4,31,32] but not for NIV [31]. The question arises how SCIRP is metabolized, and whether involved enzymes are formed inductively. Furthermore, the disappearance of SCIRP may have been influenced by the drying out of substrate. Also, the death of the fungus in such aged cultures may influence toxin amounts.

A decrease after peaking of the three scirpentriol toxins may occur also in the field. If the time pattern of 4,15-DAS, 15-MAS and SCIRP in grains is principally the same under natural as well as *in vitro* conditions, this may be at least one of the reasons behind the graded occurrence of these toxins at harvest. Thus, using methods with a detection limit <30 µg/kg, DAS, MAS and SCIRP were found in 1.7%, 2.7% and 11.4% of a total of 781, 235 and 306 oat samples respectively collected from several European countries [3]. The graded occurrence of 4,15-DAS, 15-MAS and SCIRP at the time of sampling was even more pronounced in 17 oat samples from Germany, with incidences at 0%, 29% and 53 %, respectively [17]. According to the present study, this grading may have resulted from the early appearance and disappearance of 4,15-DAS and in part also of 15-MAS, as well as from the marked delay in the formation and decrease of SCIRP up to harvest. Different rates of formation due to genetic and environmental factors have been described. Thus, the production of DAS and MAS by strains *of F. sporotrichioides* in a liquid medium absorbed into vermiculate, investigated during an incubation of 20–30 days with five day intervals, varied considerably with the strain tested and also, in part, with the incubation temperature (25 or 15 °C) [29]. Overall, it can be concluded that, in grain samples collected at harvest, the analysis of SCIRP should be considered in addition to the analysis of 4,15-DAS and 15-MAS. 

Carboxylesterases are described for *Fusarium* species as a heterogenous group consisting of several isoenzymes [29]. They are considered to be position specific by Udell and Dewick [33]. The regio-selectivity in microbial modification of trichothecenes was also demonstrated by Yoshizawa and Morooka [34] and Desjardins [18]. With regard to the formation of trichothecenes under natural conditions, the participation of plant esterases has been discussed [4,9,35]. In the present study, the substrate oats were autoclaved for 20 min prior to inoculation. Therefore, a participation of oat enzymes in the interconversion of the three scirpentriol toxins might be disregarded. 

In studies of plant toxicity using selected trichothecenes, those trichothecenes having acetylated R-groups at the carbon-3 position of the trichothecene, are less phytotoxic than their non-acetylated version [36], whereas acetylation at carbon-4 and -15 positions generally increased phytotoxicity [37]. Thus, using an *Arabidopsis thaliana* leaf assay, LD_50_ values for SCIRP, 15-MAS and 4,15-DAS were >100, 3.7, and 1.5 µM, respectively [37]. Likewise, using animal systems, an increase in toxicity from SCIRP to 15-MAS and 4,15-DAS has been found [38,39]. Therefore, the toxicity of a naturally contaminated substrate due to scirpentriol toxins possibly varies in the field due to changes in the spectrum of these toxins.
